# DpdtbA-Induced Growth Inhibition in Human Esophageal Cancer Cells Involved Inactivation of the p53/EGFR/AKT Pathway

**DOI:** 10.1155/2019/5414670

**Published:** 2019-07-01

**Authors:** Zhuo Wang, Cuiping Li, Yongli Li, Xingshuang Guo, Zhaoyu Yan, Fulian Gao, Changzheng Li

**Affiliations:** ^1^School of Basic Medical Sciences, Xinxiang Medical University, Xinxiang, Henan 453003, China; ^2^School of Basic Medical Sciences, Sanquan College of Xinxiang Medical University, Xinxiang, Henan 453003, China; ^3^Experimental Teaching Center of Biology and Basic Medical Sciences, Sanquan College of Xinxiang Medical University, Xinxiang, Henan 453003, China

## Abstract

Esophageal cancer (ESC) is one of the most deadly diseases for human. p53 in most cancers, including ESC cell, is mutated, and the mutated p53 losses its original function and acquires “gain of function” that allows for promoting the hallmarks of cancer, such as antiapoptosis, metastasis, invasion, angiogenesis, and resistance to chemotherapy. Targeting p53 through either introducing wild-type or degrading mutated p53 is an important strategy in cancer therapy. Di-2,2′-pyridine ketone dithiocarbamate s-butyric acid (DpdtbA) has significant growth inhibition against gastric cancer lines in previous study. Similar action in ESC cell lines but a novel molecular mechanism was observed in the present study. The results showed that DpdtbA exhibited an excellent antiproliferative effect for ESC cell lines (IC_50_ ≤ 4.5 ± 0.4 *μ*M for Kyse 450, 3.2 ± 0.6 *μ*M for Kyse 510 cell, and 10.0 ± 0.6 *μ*M for Kyse 150) and led to cell cycle arrest at the S phase which correlated to CDK2 downregulation. The mechanistic study suggested that growth inhibition was related to ROS-mediated apoptosis, and ROS production was due to SOD inhibition initiated by DpdtbA rather than occurrence of ferritinophagy. In addition, DpdtbA also induced a downregulation of EGFR, p53, and AKT, which hinted that mutant p53 still played a role in the regulation of its downstream targets. Further study revealed that the downregulation of p53 was through stub1- (chip-) mediated autophagic degradation rather than MDM2-mediated ubiquitination. Taken together, the DpdtbA-induced growth inhibition in a mechanism was through inactivating the p53/EGFR/AKT signal pathway.

## 1. Introduction

Cancer is one of most deadly diseases, while esophageal cancer (ESC) lies in the eighth position in cancer-caused deaths, and about half of all cases occur in China [1]. Despite advancements in diagnostic and treatment methods in recent years, the prognosis of patients with ESC remains not ideal [2]. Although many factors may induce the formation of esophageal cancer, the underlying mechanism of it is largely unknown. p53 is one of most important transcription factor, regulating proliferation, apoptosis, autophagy, and cell cycle, and normally is considered as a tumor suppressor gene. However, p53 in human cancer is most frequently mutated and in dominant phenotypes, in human ESC, 75% of p53 gene mutations were detected [3]. And mutant p53 (mutp53) cancers are dependent on their hyper stable mutp53 protein for survival [4]. In addition, evidences revealed that mutp53 seemed to gain characteristics that allow for promoting the hallmarks of cancer, such as antiapoptosis, metastasis, invasion, angiogenesis, and resistance to chemotherapy [5], which actively contribute to cancer development and progression [5, 6]. p53 mutants are categorized into structural (R175, G245, R249, and R282) and contact (R248 and R273) mutations with effects of gross conformational alterations and loss of anchorage to DNA, respectively [7]. Generally, wild-type p53 is regulated by its gene expression and degradation and is maintained at a low level by continuous degradation via proteasome through E3 ubiquitin ligase [8, 9], others, such as chaperone-mediated autophagic degradation [10–14]. As mentioned above, since different mutant p53 alleles may exhibit certain unique characteristics in cancer development and progression, therefore, targeting mutant p53 for protein degradation, rather than its reactivation, might be another strategy for drug development [15]. Garufi et al. reported that a Zn(II)-curcumin complex displayed growth inhibition involved in the induction of mutant p53 degradation [16], implying that mutant p53 degradation was one of the options in cancer treatment. However, the exploration of effective small molecule therapies targeting mutp53 is still on the way.

The epidermal growth factor receptor (EGFR), an ErbB family of receptor tyrosine kinases, plays an important role in cell proliferation and survival [17]. Upon ligand binding, the EGFR dimerizes which lead to subsequent activation of EGFR tyrosine kinase, resulting in the generation of a number of intracellular signals, including PI3K/AKT/mTOR, RAS/MAPK1/3, and STAT3 signaling pathways [18]. Overexpression of EGFR is seen in many solid tumors, including esophageal cancer, and is associated with poor prognosis [19]. In addition, the frequent gene amplification of EGFR, HER2, and FGFR2 and the presence of active EGFR mutations were observed in ESC specimens [20]. Downregulation of EGFR or inhibition of EGFR kinase may halt the proliferation of cancer cells; thus, many tyrosine kinase inhibitors such as gefitinib, lapatinib, and erlotinib have been developed; however, only limited effectiveness were achieved [21]. It was reported that some small molecules could downregulate EGFR and enhance the effectivity of chemotherapeutic agent [22]. Thus, the development of small-molecule inhibitors for the treatment of esophageal cancer is required.

As mentioned above, different mutant p53 alleles play an important role in cancer development and progression; therefore, targeting mutant p53 through protein degradation, rather than its reactivation, might be another strategy for drug development. Dithiocarbamates as metal chelators own important biological activities in the treatment of bacterial and fungal infections, AIDS, and cancer [23]. DpdtbA (di-2,2′-pyridine ketone dithiocarbamate s-butyric acid), a dithiocarbamate derivative, also displayed significant growth inhibition against gastric cancer cell lines in our previous study [24]. To extend our knowledge for the dithiocarbamate derivative, the effect of DpdtbA on the proliferation of esophageal cancer cell lines was further investigated. Interestingly, the DpdtbA-induced growth inhibition involved p53 depletion, which was not consistent with that reported previously in gastric cancer cell lines [24]. Additional investigations revealed that the p53 degradation was through chaperon-mediated autophagy rather than MDM2-mediated ubiquitination. Furthermore, concomitant to the degradation of mutated p53, a downregulation of EGFR and AKT was observed, indicating that inactivation of the p53/EGFR/AKT axis could achieve the growth inhibition in p53 mutation-overexpressed ESC cell lines. Those results definitely enriched our knowledge that targeting mutant p53 may be one of the options in successful anticancer therapy.

## 2. Results

### 2.1. DpdtbA Induced Proliferation and Colony Formation Inhibition in Esophageal Cancer (ESC) Cells

**Figure 1:**
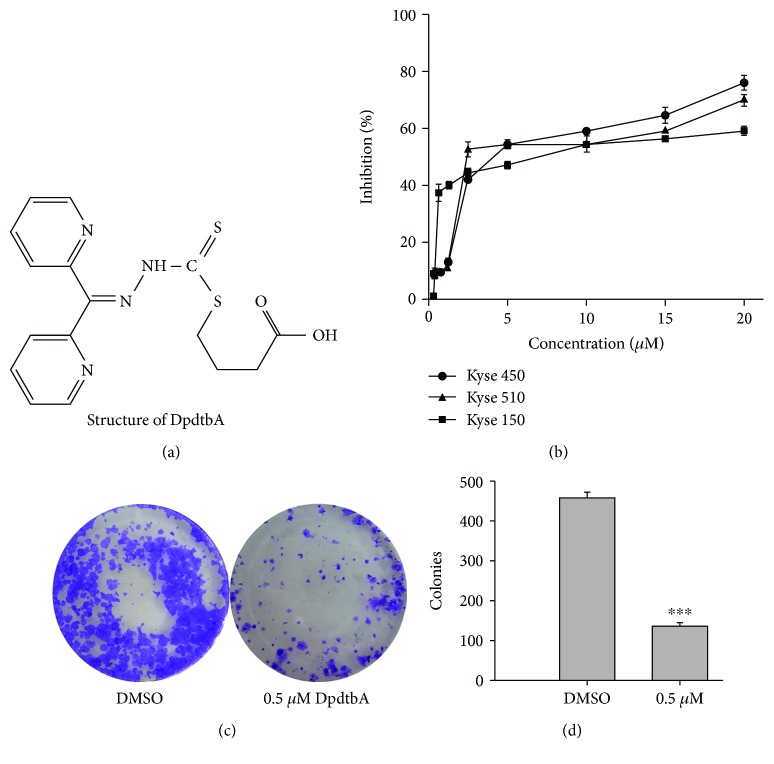
DpdtbA induced growth and colony formation inhibition. (a) Structure of DpdtbA; (b) the effect of DpdtbA on the proliferation of ESC cell lines; (c) DpdtbA displayed colony formation inhibition; (d) quantitative analysis of alteration in colony numbers (from trice measurements). ^∗∗∗^ *p* < 0.05.

Previous study demonstrated that di-2,2′-pyridine ketone dithiocarbamate s-butyric acid (DpdtbA) owned significant growth inhibition in gastric cell lines [24]; DpdtbA might have similar action in ESC cell lines. With this purpose, we first assessed the effect of DpdtbA on the cell viability of Kyse 450, 150, and 510 cells. The dose-response curves are depicted in Figure 1, and significant growth inhibition for the ESC cell lines (IC_50_ ≤ 4.5 ± 0.4 *μ*M for Kyse 450, 3.2 ± 0.6 *μ*M for Kyse 510, and 10.0 ± 0.7 *μ*M for Kyse 150) was observed compared to control (*p* < 0.05), but the cell line dependence was not evident. Next, the effect of DpdtbA on cell colony formation was further investigated. As shown in Figure 1(c), DpdtbA induced a significant reduction in colony numbers and populations for Kyse 450 (*p* < 0.05); the quantitative analysis is shown in Figure 1(d). Similar assay for Kyse 150 was also performed, and the results are presented in Figure S1.

### 2.2. DpdtbA Induced Cell Cycle Arrest at the S Phase

**Figure 2:**
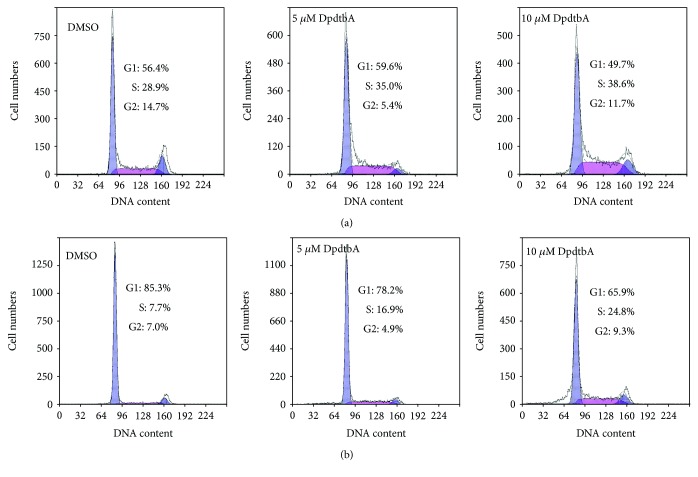
Effect of DpdtbA on cell cycle in ESC cells. Cell cycle distribution of ESC cells following treatment with various concentrations of DpdtbA. (a) Kyse 450 cells and (b) Kyse 150 cells; dose-dependent accumulation in the S phase of the cell cycle. Accordingly, the proportions of cells in the G1 and G2/M phases were decreased.

To test whether an induction of cell cycle arrest contributed to the antiproliferative capability of DpdtbA in ESC cells, cell cycle analysis was performed via flow cytometry. As shown in Figure 2, DpdtbA caused an accumulation of the ESC cells in the S phase for both cell lines, and the percentages at the S phase significantly increased by 10 to 17% during 24 h insult of the agent, thereby decreasing the proportion of cells in the G1 phase. Those indicated that DpdtbA could disturb cell cycle and arrest the cells at the S phase, which was not consistent with that in gastric cell lines [24], indicating that DpdtbA-induced cell cycle delay was cell line dependent. Furthermore, it was well documented that the progression of cells is regulated by cyclins and CDK (cyclin-dependent kinase) proteins, and cyclin A and CDK2 are known to play an important role in the regulation of DNA synthesis during cell-cycle progression at the S phase; thus, the expression of CDK2 in different conditions was determined. As shown in Figure S2, DpdtbA led to a downregulation of CDK2, which contributed to S phase arrest, in accordance with that reported previously [25, 26].

### 2.3. The DpdtbA Induced Significant Apoptosis in ESC Cells

**Figure 3:**
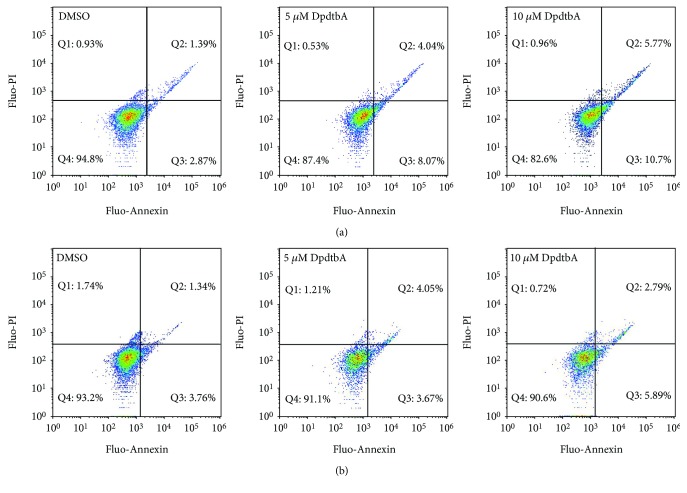
Apoptosis analysis of ESC cell lines via flow cytometer. DpdtbA was incubated with the cells for 24 h. All attached cells were collected and double stained with annexin V and propidium iodide (PI) using a kit from Dojindo Laboratories following the manufacturer's instructions. (a) Kyse 450 cells and (b) Kyse 150 cells. The condition was as indicated in the figure.

Previous study revealed that DpdtbA-induced apoptosis contributed to the growth inhibition in gastric cancer lines [24]; similar action may occur in ESC cells. To this end, the ESC cells were pretreated by DpdtbA; then, the annexin V/propidium iodide (PI) staining was performed to measure the apoptotic populations at early and late stages, which were achieved by monitoring the externalization of phosphatidylserine on the cell surface of apoptotic cells. The results from flow cytometric analyses showed that the DpdtbA induced early apoptosis and late apoptosis in a concentration-dependent manner (Figure 3(a), from 4.2 to 16.4% for Kyse 450 and 5.1 to 8.1% for Kyse 150). Statistical analysis revealed that the apoptotic induction of DpdtbA at a concentration of 5 and 10 *μ*M had a statistical significance for Kyse 450 (*p* < 0.05), but for Kyse 150 cells, 10 *μ*M was required (*p* < 0.05). Moreover, the apoptotic portions in both cell lines were obviously different, which may be relative to IC_50_ value; as a whole, DpdtbA induced a limited apoptosis.

To seek additional evidence for the occurrence of apoptosis, the variations in nuclear morphology and fragmentation of chromosomal DNA were further investigated. As shown in Figure S3, the condensed nuclear and fragmentation of chromosomal DNA were observed upon DpdtbA treatment. In addition, using AO/EB stains to detect apoptosis was also performed under fluorescence microscope [27, 28]. As shown in Figure S4, live cells appeared uniformly green and had intact membrane and uniform chromatin, whereas early apoptotic cells and late apoptotic cells appeared as bright green and orange, respectively; necrotic cells appeared as red with no condensed chromatin. The portions of apoptosis cells in Kyse 450 were higher than that of Kyse 150, consistent with the results from annexin V/PI stains (Figure 3).

**Figure 4:**
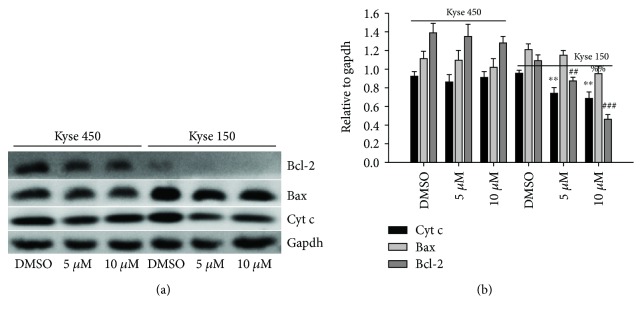
The alteration in apoptosis-related genes after DpdtbA treatment in the ESC cells. (a) Western blotting analyses of apoptosis-related gene expressions. (b) Quantitative analyses of the levels of apoptosis-related genes in the presence or absence of DpdtbA (from twice measurements). ^∗∗^^,##,%%^ *p* < 0.05 and ^###^ *p* < 0.01.

Bcl-2 family members play key roles in the regulation of apoptotic progress. To understand how DpdtbA induced apoptosis, we further examined the alteration in the expression of apoptosis-related genes in ESC cells. As shown in Figure 4, the DpdtbA treatment led to slight downregulation of the bcl-2 level, but the expression of bax was not increased, and similar situation occurred for cytochrome c. It was well documented that translocation of bax on mitochondria could lead to alteration in mitochondrial membrane permeability (MMP). Since there was no obvious change in the bax expression, the MMP could be in an intact state. To confirm above speculation, a mitochondrial dye, rhodamine 123, was employed to evaluate the permeability of mitochondrial membrane. As expected, the MMP has almost no change (Figure S5), supporting that a limited apoptosis occurred.

### 2.4. The ROS Production Stemmed from DpdtbA Induced SOD Inhibition Rather Than Ferritinophagy

**Figure 5:**
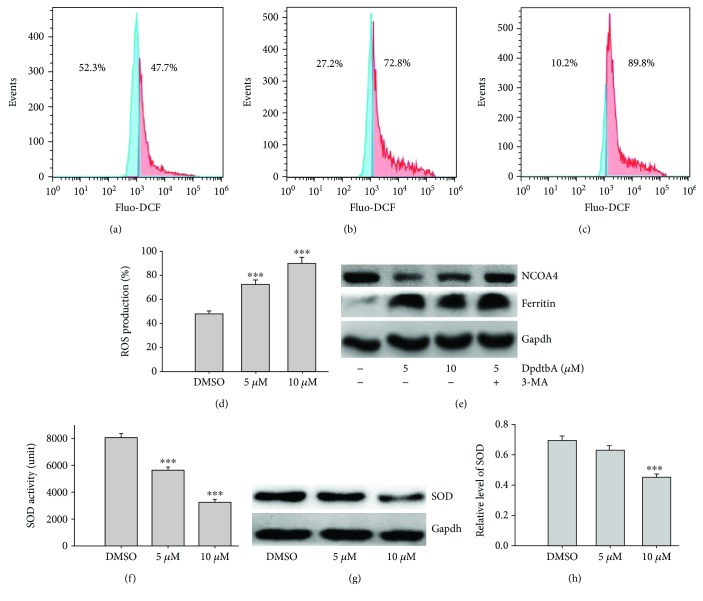
DpdtbA induced ROS generation. Flow cytometry analysis from Kyse 450 cells stained by DCF: (a) DMSO; (b) 5 *μ*M DpdtbA; (c) 10 *μ*M DpdtbA; (d) quantitative analysis of ROS production (from twice measurements); (e) western blotting analyses of ferritinophagy-related proteins, the condition was as indicated; (f) the effect of DpdtbA on SOD activity (from trice measurements); (g) alteration of the SOD expression in the absence or presence of DpdtbA; (h) quantitative analysis of alteration in the level of SOD (from twice measurements). ^∗∗∗^ *p* < 0.01.

In general apoptosis associated with ROS production, to determine the origin of ROS, the ESC cells treated by DpdtbA were stained by ROS dye, DCF, following flow cytometry analysis. The ROS production at different condition is shown in Figure 5. Compared to control (Figure 5(a)), the ROS production induced by DpdtbA (Figures 5(b) and 5(c)) was significantly increased (*p* < 0.05, Figure 5(d)) in Kyse 450, indicating that ROS indeed involved the action of DpdtbA. Similar result was obtained in Kyse 150 cells (Figure S6). Due to the diversity in ROS production, the possible site was further explored. Ferritinophagy is an important contributor in Fenton reaction-related ROS generation; thus, the level of ferritin and its specific cargo, NCOA 4, was assayed. Unexpectedly, DpdtbA did not induce ferritinophagy that led to ferritin degradation; contrarily, an upregulated ferritin was observed with downregulated NCOA4 (Figure 5(e)). Furthermore, addition of autophagy inhibitor (3-MA) did not alter the status of ferritin and NCOA4, those excluded that ROS production was through ferritinophagy, which was different from other iron chelator [29]. Moreover, the status of ROS in a cell was dependent on the balance between ROS production and antioxidant system. The alterations of antioxidants, such as GST and superoxide dismutase (SOD), significantly affect the status of oxidative stress. The risen ROS might be from downregulated SOD or SOD inhibition. To this end, the level of SOD was investigated, as shown in Figure 5(g); DpdtbA led to downregulation of SOD with a significant difference compared to control (*p* < 0.01 at 10 *μ*M, Figure 5(h)). Meanwhile, DpdtbA was also able to inactivate SOD in a concentration-dependent manner (*p* < 0.01, Figure 5(f)). Taken together, the ROS production during DpdtbA insult mainly stemmed from inactivation and downregulation of SOD.

### 2.5. The DpdtbA Induced Growth Inhibition Correlated to EGFR Downregulation

**Figure 6:**
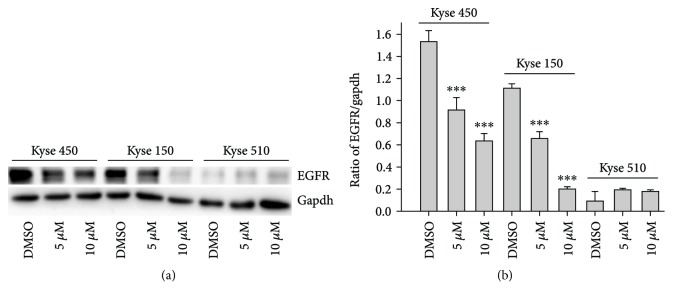
DpdtbA led to EGFR downregulation in the indicated ESC cell lines: (a) western blotting analysis and (b) quantitative analysis of alteration of EGFR in the presence or absence of DpdtbA (from twice measurements). ^∗∗∗^ *p* < 0.01.

It has shown that EGFR mediates cell proliferation [30], and generally, EGFR (wild-type or mutated) is overexpressed in ESC cells [18]. Whether the growth inhibition induced by DpdtbA was associated with alteration of EGFR, with this purpose, we determined the expression of EGFR in the presence or absence of DpdtbA. As shown in Figure 6, the expression of EGFR in Kyse 450 and Kyse 150 cell lines was decreased, but less abundance of EGFR in Kyse 510 cells was observed. The quantitative analysis of EGFR revealed that DpdtbA induced downregulation of EGFR had a statistical significance (*p* < 0.01), hinting that depletion of EGFR might involve in the growth inhibition. Due to lower abundance of EGFR in Kyse 510, the cell line was further not included in the following investigation.

### 2.6. The Effect of DpdtbA on the Upstream and Downstream of EGFR Signal

**Figure 7:**
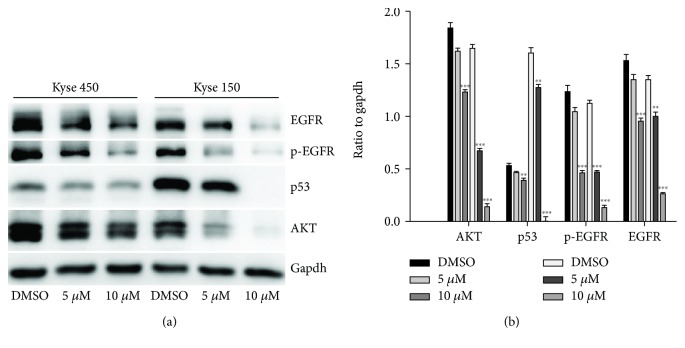
Alteration of EGFR was associated with the change of upstream and downstream targets. (a) Western blotting analysis; (b) quantitative analyses of alteration in the p53/EGFR/AKT signal pathway before and after DpdtbA treatment (from twice measurements). ^∗∗^ *p* < 0.05, ^∗∗∗^ *p* < 0.01.

It has known that EGFR activation can trigger the alteration of others, including JAK/STAT and the PI3K/AKT signal pathway, thus promoting cell growth [30]. The fact that DpdtbA induced a downregulation of EGFR prompted us to explore the underlying mechanism. Thus, the regulation of EGFR on other genes was further investigated. Generally, AKT was regulated by EGFR (or phosphorylated EGFR); the alteration of EGFR might also affect its downstream target, AKT; thus, the level of AKT in the presence or absence of DpdtbA was determined. As expected, accompanied by a decrease of EGFR (or p-EGFR), the AKT was also downregulated (Figure 7(a)). The quantitative analysis indicated that DpdtbA induced the decrease of AKT which had a statistical significance (*p* < 0.01) in both cell lines, indicating that EGFR was involved in the regulation of AKT. In addition, it is well documented that p53 can regulate EGFR [31]; the p53 expression, therefore, was further assayed. Interestingly, DpdtbA also led to p53 downregulation (Figure 7(a)). Statistical analysis revealed that the levels of p53, EGFR, and p-EGFR were significantly decreased after DpdtbA treatment (*p* < 0.05, Figure 7(b)), which hinted that downregulation of EGFR and AKT might stem from the downregulation of p53 (or mutant p53 might still play a role in the modulation of its downstream targets).

### 2.7. The Regulative Action of Mutant p53 on Its Downstream Target Genes and Growth Inhibition

**Figure 8:**
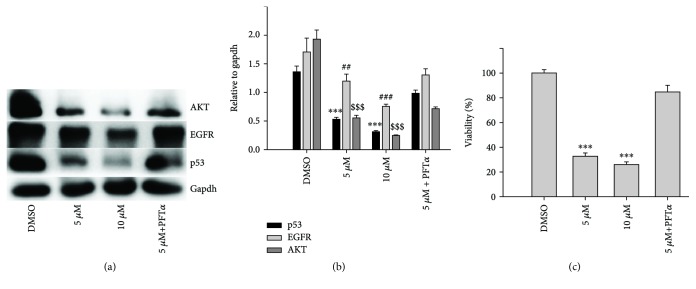
p53 downregulation was associated with EGFR and AKT downregulation. (a) Western blotting analysis in the given condition; (b) quantitative analyses of alteration of EGFR, p53, and AKT in the presence or absence of PFT-*α* (from twice measurements). (c) DpdtbA-induced growth inhibition correlated with p53 downregulation. And the quantitative analysis was based on quartic measurements. ^##^ *p* < 0.05, ^∗∗∗^^,###,$$$^ *p* < 0.01.

As mentioned above, DpdtbA treatment led to downregulation of p53 at the protein level in both Kyse 450 and Kyse 150 cell lines, which could be achieved through modulation in transcription and in translation. To corroborate that p53 downregulation might be through a posttranslational modification instead of transcriptional modulation, the alteration of p53 at the mRNA level was determined via RT-PCR. As shown in Figure S7, DpdtbA could upregulate p53 in transcription, which was not consistent with p53 downregulation at the protein level, hinting that p53 downregulation was not through transcriptional regulation. This was not surprising for p53 was a stress protein in response to different insulting. Next, the regulation of p53 required to be further determined. There is convincing evidence from reporter assays that mutant p53 has the ability to transactivate specific target genes, such as c-Myc and EGFR promoter in a manner distinct from wild-type [32, 33]. To determine whether the mutant p53 (H179R in Kyse 450, R248Q and R155Q in Kyse 150) could regulate its downstream target genes, the p53 inhibitor, PFT-*α* was used to confirm the action of p53. As shown in Figure 8, DpdtbA induced downregulation of p53 in a concentration-dependent manner, but addition of PFT-*α* impaired the DpdtbA-induced p53 degradation; PFT-*α* alone did not significantly modify p53 levels (Figure 8), in accordance with the result reported previously [16]. And interestingly, similar situation occurred for both EGFR and AKT, supporting that the mutated p53 played a role in downstream gene regulation in the ESC cell lines. Furthermore, quantitative analysis was given in Figure 8(b); clearly, the alteration in the p53/EGFR/AKT axis had a significant difference before and after DpdtbA treatment (*p* < 0.05), but the additional effect was not observed in the combination treatment, which might be due to the difference in interaction between PFT-*α* and mutant and wild-type p53 [34, 35]. To corroborate that the depletion of p53 may contribute to the growth inhibition in ESC cells, the effect of PFT-*α* combination with DpdtbA on proliferation was further investigated; Figure 8(c) clearly showed that PFT-*α* attenuated DpdtbA-induced growth inhibition (PFT-*α* has a very weak effect on ESC cell growth, data not shown), in accordance with the result from western blotting. However, the addition of (NH_4_)_2_Fe(SO_4_)_2_ blocked the action of DpdtbA on both growth inhibition and downregulation of p53 (Figure S7), hinting that the induction was dependent on the chelating status of DpdtbA.

### 2.8. The p53 Depletion Involved stub1 Chaperone-Mediated Autophagy Rather Than MDM2-Mediated Ubiquitination

**Figure 9:**
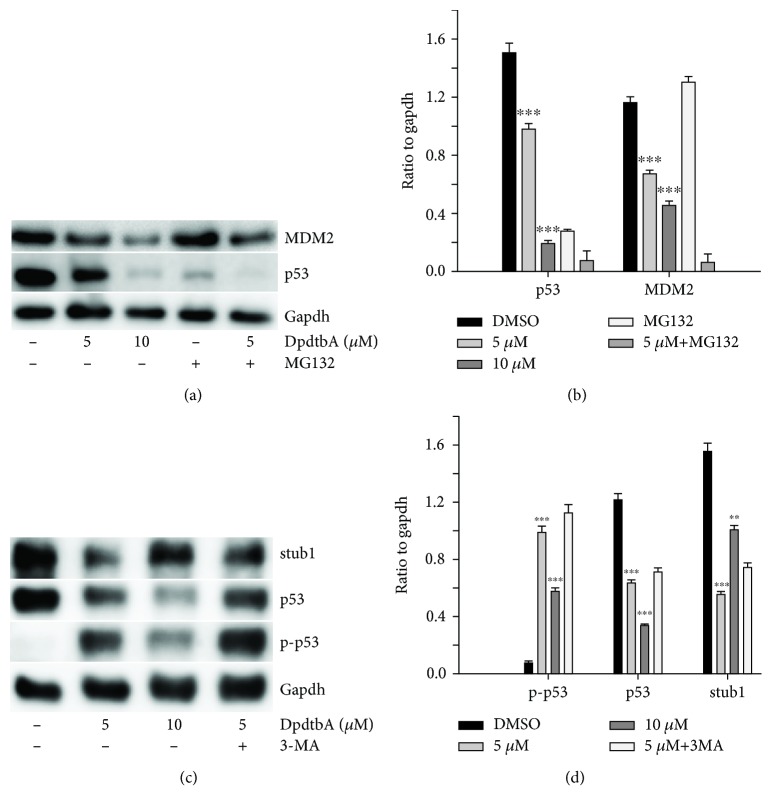
DpdtbA induced p53 deletion. (a) p53 deletion did not involve ubiquitination; (b) quantitative analyses of alterations of MDM2 and p53 (from twice measurements); (c) p53 deletion might involve stub1-mediated autophagy; (d) quantitative analyses of alterations of stub1 and p53 (from twice measurements). The conditions were as indicated. ^∗∗^ *p* < 0.05, ^∗∗∗^ *p* < 0.01.

As mentioned above, DpdtbA induced p53 degradation; accordingly, its downstream target, EGFR and AKT, was also downregulated; it was suggested that the degradation of p53 was mainly a molecular event. Hence, the detail in p53 degradation required to be further determined. We then tested whether MDM2 played a role in such p53 degradation. To this end, the levels of p53 and MDM2 were assayed. As shown in Figure 9(a), both p53 and MDM2 were downregulated during DpdtbA treatment, and alterations in the gene expressions had a statistical significance (*p* < 0.05, Figure 9(b)). Moreover, such effect in downregulation of p53 could also be achieved by MDM2 knockdown via siRNA (Figure S8), indicating that the function of MDM2s E3 ligase was not involved in the ubiquitination of p53, which was further supported by the fact that addition of proteasome inhibitor, MG-132, did not attenuate the p53 degradation (Figure S9). This was in accordance with that reported previously [33]. Since MDM2 was not responsible for the degradation of mutant p53 in our setting, we then further explored the role of autophagy, a cellular mechanism of protein degradation within lysosomes. It was reported that chip (stub1) promoted autophagy-mediated degradation of aggregating mutant p53 [36]; DpdtbA-induced p53 degradation might involve this pathway. To test the hypothesis, the level of stub1 (chip) was evaluated. As shown in Figure 9(c), the alteration of stub1 was similar to that of p53 upon DpdtbA treatment, and an important observation was that p53 and stub1 were restored when addition of 3-methyladenine (3-MA), hinting that p53 degradation might be through stub1 chaperon-mediated autophagic degradation. The quantitative analysis was given in Figure 9(d); clearly, the alterations in the level of p-p53, p53, and stub1 had a statistical significance (Figure 9(d), *p* < 0.05).

### 2.9. p53 Autophagic Degradation Dominated the Alteration of Its Downstream Targets

**Figure 10:**
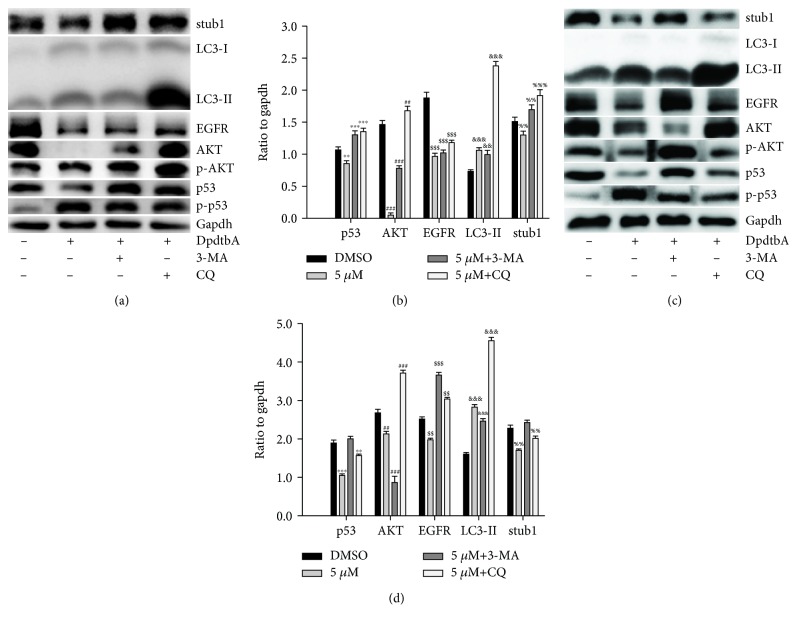
p53 autophagic degradation regulated its downstream targets, (a, b) Kyse 450 cells and (c, d) Kyse 150 cells. (a, c) Western blotting analyses of alteration in the p53/EGFR/AKT axis and stub1-mediated autophagy relative proteins, the condition was as indicated. (b, d) Quantitative analyses of alteration of the expression of the p53/EGFR/AKT axis and LC3-II as well as stub1. CQ = chloroquine. Quantitative analyses of those proteins from Kyse 450 and Kyse 150 cells were performed from twice measurements. ^∗∗^^,##,$$,&&,%%^ *p* < 0.05, ^∗∗∗^^,###,$$$,&&&,%%%^ *p* < 0.01.

Since stub1 chaperon-mediated autophagy involved the p53 degradation, accordingly, it may affect the downstream target of p53. To this end, autophagy-related genes, LC3, stub1, and along with the p53/EGFR/AKT axis were assessed. As shown in Figure 10(a), concomitant to the autophagy activation (increase in LC3-II and stub1), a downregulated p53, EGFR, and AKT were observed, and the alterations of those proteins are shown in Figure 10(b). As shown in Figure 10(a), the stub1 chaperon-mediated autophagy was clearly responsible for the p53 degradation, which further led to downregulation of its downstream targets. This conclusion was further supported by the experiment of RNA interference, because the knockdown of stub1 by siRNA could attenuate p53 depletion induced by DpdtbA (Figure S10). Furthermore, addition of autophagy inhibitor (3-MA or chloroquine) could increase protein expressions in the p53/EGFR/AKT axis, which indicated that autophagic degradation of p53 modulated the EGFR/AKT pathway. Similar result was observed in Kyse 150 cells (Figures 10(c) and 10(d)), supporting that stub1 chaperon-mediated autophagy involved the p53 degradation, and the mutant p53 still played a role in the gene regulation.

As mentioned above, DpdtbA induced growth inhibition, cell cycle arrest, and apoptosis, which involved ROS production due to SOD inhibition. Further mechanistic study revealed that DpdtbA led to stub1-mediated autophagic degradation of p53 that dominated the level of its downstream targets EGFR and AKT; thus, DpdtbA-induced growth inhibition could be through inactivating the p53/EGFR/AKT signal pathway.

## 3. Discussion

Esophageal cancer (ESC) is one of the most deadly diseases, and the long-term survival of the patients is poor [15]; therefore, new therapeutic strategy is required. It is well documented that iron is an essential element and plays a crucial role in cellular proliferation and DNA synthesis. Compared to normal cells, neoplastic cells have a high requirement for iron for their growth. *In vivo* neoplastic cells can obtain iron from local environment, i.e., tumor microenvironment which is comprised of various cells, cytokines, and extracellular matrix; among those, tumor-associated macrophages (type II phenotype) are the main source of iron supply [37]. Obviously, iron isolation or chelation from tumor environment may help to inhibit the growth of tumor cells. Dithiocarbamates had showed a significant antiproliferative action against gastric cancer cell lines in our previous study [24], which prompted us to extend additional investigation to different cancer cell lines, such as ESC cell lines, in order to gain more knowledge for the agent (Figure 1). Disturbing cell cycle is often found in the mechanism for many chemotherapeutic agents. Iron chelator induced growth inhibition through inhibiting ribonucleotide reductase, accordingly depleting dNTPs and resulting in S phase arrest [38]. In addition, iron depletion also led to alteration of CDKs, resulting in a decrease of CDK2 in human T lymphocytes [39]. Similarly, the DpdtbA also induced ESC cell accumulation at the S phase (Figure 2) and a decrease of CDK2 (Figure S1). In addition, ROS production normally is involved in the action of mechanism for most chemotherapeutic drugs, and the ROS can be generated through Fenton reaction, dysfunction of mitochondria, or imbalance of the redox system, which result in apoptosis and autophagy. Therefore, we further evaluated ROS and identified the source of ROS production. As shown in Figure 5(a), DpdtbA-induced growth inhibition was involved in ROS production; however, ROS production is not due to mitochondrial and lysosomal dysfunction but to SOD inhibition. It has been reported that some iron chelators can induce ferritin degradation in lysosome, which lead to an increase of iron in LIP [29, 40], but in our study, DpdtbA did not induce ferritin degradation (Figure 5(d)). The analysis of integrity of the mitochondrial membrane eliminated the alteration of mitochondrial membrane permeability for the bax and cytochrome c were not upregulated. So we deduced that the excess ROS might be due to imbalance in the antioxidant-oxidant system. It is well known that superoxide dismutase (SOD) as an antioxidant is responsible for the decomposition of superoxide, and copper and zinc ion as cofactors are located in its catalytic center. DpdtbA as metal chelator may influence SOD activity. Our data revealed that DpdtbA both inactivated SOD activity and also led to downregulation of SOD (Figures 5(f) and 5(g)), resulting in the imbalance of the redox system. Furthermore, excessive ROS production led to occurrence of apoptosis; the results both from flow cytometric analysis and from AO/EB staining (Figure 3 and Figure S4) demonstrated that DpdtbA could induce the occurrence of apoptosis, but the susceptibility of apoptosis induction for the investigated cell lines was a slight difference; Kyse 450 cells seem to be more prone to induce apoptosis than Kyse 150 cells. We speculated that the difference in apoptosis induction may relate to the status and abundance of mutation of p53 [41], while the prosurvival effect of bcl-2 might retreat to the back of the p53, playing a secondary role in response to chemotherapeutic agent because mutant p53 (mutp53) cancers are dependent on their hyper stable mutp53 protein for survival (Figures 4 and 7) [4]; this might endow Kyse 150 cell less sensitive than Kyse 450 cell. However, the other signal pathway may also play a critical role in growth inhibition and apoptosis induction.

Overexpression of epidermal growth factor receptor (EGFR) occurs in approximately 80% of patients with adenocarcinoma and squamous cell carcinoma [42], and many studies have demonstrated that overexpression of EGFR is associated with a lower survival rate [43, 44]. Thus, the epidermal growth factor receptor (EGFR) family is receiving considerable attention. Small molecule tyrosine kinase inhibitors and monoclonal antibodies have been explored in patients with esophageal cancers; however, only a modest clinical activity is achieved. The depletion of EGFR might be other option in esophageal cancer treatments. In the present study, we illustrated that DpdtbA could downregulate EGFR in ESC cell lines (Figure 6), which forced us to consider whether the alteration of EGFR stemmed from the alteration of its upstream molecule, p53. As expected, DpdtbA treatment led to downregulation of p53, which confirmed that the p53/EGFR pathway still activated in ESC cancer cells. Furthermore, DpdtbA also caused downregulation of AKT, a downstream molecule of EGFR, indicating that DpdtbA could inactivate the p53/EGFR/AKT pathway, in accordance with the observation from literature [45]. It is well documented that p53 is often mutated and overexpressed in cancer cells, restoring p53 function; reintroducing or rescuing wild-type p53 into cancer cells could achieve inhibition of cancer [46, 47]. On the other hand, the accumulating evidences reveal that stabilization of mutant p53 in tumors is important for its oncogenic activities; thus, depletion of mutant p53 may attenuate the malignant properties of cancer cells [48]. Generally, wild-type p53 is modulated by proteasomes, and MDM2, a p53 specific E3-ubiquitin-protein ligase, plays an important role in p53 homeostasis. However, the mutant p53 degradation was not through the MDM2 pathway; similar result was obtained in the present study for downregulation of MDM2 by siRNA which did not restore the p53 level. Autophagy is an important proteolytic system to devote to clearing cellular misfolded proteins or protein aggregates [49], and moreover, it was also reported that chip promotes autophagy-mediated degradation of aggregating mutant p53 [33]; we speculated that the mutant p53 degradation might involve autophagy. stub1 (chip) has been shown to be important for mutant p53 degradation both in normoxia and in hypoxia [15, 50–52]; the stub1 (chip) might also involve the p53 degradation induced by DpdtbA. As expected, the stub1 was downregulated with the decrease of p53 when DpdtbA was exposed to the cells (Figure 9), and knocking down of stub1 by siRNA intervention led to downregulation of p53, which supported that stub1 mediated the degradation of p53 (Figure S10). In addition, with the decrease of p53, the level of microtubule-associated protein light chain 3 (LC3-II) increased, indicating that the degradation of p53 involved autophagy (Figure 10); therefore, autophagic degradation of p53 determined the level of EGFR/AKT. Those indicated that mutant p53 (H179R) (or R248Q and R155Q in kyse150) in Kyse 450 cells still played a role in gene regulation, in accordance with the findings from other laboratories [53], such as Dong et al., who demonstrated that the expression of p53 gain-of-function mutation R175H in endometrial cancer cells increased the invasive phenotypes by activation of the EGFR/PI3K/AKT pathway [33]. In addition, some mutants of p53 (G245C and R273H) in esophageal squamous cells confer a stronger proliferative capacity [54]. Those demonstrated that depletion of mutant p53 is one of the important strategies in cancer therapy. DpdtbA-induced growth inhibition in the ESC cells was through inactivation (or degradation) of the p53/EGFR/AKT signal pathway, providing additional example to strengthen this concept in cancer therapy as like other chemotherapeutic agents [55].

In conclusion, DpdtbA-induced growth inhibition involved apoptosis and cell cycle arrest. Further study revealed that ROS production involved the apoptosis induction, and the rising ROS was stemmed from SOD inhibition initiated by DpdtbA rather than occurrence of ferritinophagy. Furthermore, DpdtbA could induce downregulation of EGFR, p53, and AKT, hinting that the mutant p53 still played a role in the proliferation of ESC cells. Additional study revealed that stub1- (chip-) mediated autophagy was responsible for the p53 degradation rather than MDM2-mediated ubiquitination, which inactivated the EGFR/AKT signal pathway. Taken together, DpdtbA-induced growth inhibition was through inactivating the p53/EGFR/AKT signal pathway.

## 4. Materials and Methods

### 4.1. Materials

MTT, 3-methyladenin (3-MA), pifithrin-*α* (PFT-*α*), RPMI-1640, and other chemicals were purchased from Sigma-Aldrich. Gapdh, NCOA4, Bcl-2, and AKT antibody were obtained from EnoGene (Nanjing, China); antibodies EGFR, stub-1, SOD, MDM2, Cyt-C, LC3, Bax, and p-AKT were purchased from Proteintech Group Inc. (Wuhan, China). Antibodies p53, p-p53, and ferritin were purchased from Cell Signaling Technology (Massachusetts, USA); siRNA for MDM2 (stB0001232) and stub1 (stB0001238) were obtained from RiboBio (Guangzhou, China).

### 4.2. Cytotoxicity Assay (MTT Assay)

A 10 mM DpdtbA (di-2,2′-pyridine ketone dithiocarbamate s-butyric acid) in 70% DMSO was diluted to the required concentration with DMSO. The MTT assay was conducted as previously described [24]. The Kyse 450, Kyse 150, and Kyse510 cell lines (Cell Resource Center, Institute of Basic Medicine, Chinese Academy of Medical Sciences, China) were used in the current investigation. According to the Catalogue of Somatic Mutations in Cancer (COSMIC) database (http://cancer.sanger.ac.uk/cancergenome/projects/cosmic/), the status of TP53 is H179R in Kyse 450, R248Q and R155Q in Kyse 150, and nonmutation in Kyse 510. Briefly, the Kyse 450 cells (5 × 10^3^/ml) were seeded equivalently into a 96-well plate, and the various concentrations of DpdtbA were added after the cells adhered. Following 48 h incubation at 37°C in a humidified atmosphere of 5% CO_2_, 10 *μ*l MTT solution (5 mg/ml) was added to each well and additional incubation applied. Finally, 100 *μ*l DMSO was added in each well to dissolve the formed formazan after removing the cell culture. The absorption of the solution that was related to the number of live cells was measured on a microplate reader (MK3, Thermo Scientific) at 490 nm. Percent growth inhibition was defined as percent absorbance inhibition within appropriate absorbance in each cell line. The same assay was performed in triplet.

### 4.3. Plate Clone Formation Assay

The cells in the exponential phase were trypsinized and seeded in 6-well plates at the density of 500 cells/well. The cells were kept in sustaining insulting of DpdtbA at a dose of 1/20 IC_50_ or 1/10 IC_50_. Fourteen days later, colonies were fixed in 3.7% paraformaldehyde and stained with 0.1% crystal violet. Colonies containing 50 cells at least were counted under inverse microscope (Nikon, Tokyo, Japan), and the clone numbers were analyzed subsequently.

### 4.4. Flow Cytometry Analysis of Apoptosis and Cellular ROS

Cellular ROS determination was performed based on previously described [29]. Apoptosis was measured using the Apoptosis Detection Kit (Dojindo Laboratories) as the company recommended. Briefly, Kyse 450 (150) cells were treated with DpdtbA for 24 h. Following this, cells were collected, washed, and stained with annexin V-FITC and propidium iodide (PI) following the manufacturer's instruction (Dojindo Laboratories, Japan). The intracellular ROS assay was similar to the abovementioned protocol, except that H_2_DCF-DA was used to stain the cells.

### 4.5. Cell Cycle Analysis

The Kyse 450 cells (or Kyse 150 cells) (1 × 10^5^) were seeded in a 6-well plate and incubated for 24 h at 37°C (5% CO_2_). The medium was replaced with fresh medium supplemented or not (control) with DpdtbA (5 and 10 *μ*M). Following 24 h of incubation, the cells were collected with centrifugation, washed with PBS, finally fixed in 70% ethanol, and stored at -20°C. After removing the 70% ethanol and washing with PBS, the cellular nuclear DNA was stained based on the company recommended protocol; the cells in staining buffer were directly subjected to flow cytometer analysis. For each sample, 10,000 events were collected, and fluorescent signal intensity was recorded and analyzed by CellQuest and ModiFit (Becton Dickinson, USA).

### 4.6. SOD Activity Assay

The SOD activity was determined as the company recommended. Briefly, the Kyse 450 cells were treated with DpdtbA for 24 h, then collected and lysed. The supernatant was separated by centrifugation at 4°C, the protein concentration was determined by a colorimetric Bio-Rad DC protein assay on a microplate reader MK3 at 570 nm. Next, the same amount of protein mixture was mixed with WST-8/enzyme solution and initiator solution, following incubation for 30 min. at 37°C; the absorbance at 450 nm was measured on the abovementioned microplate reader. The assay of SOD activity followed similar protocol; the DpdtbA was directly added to solution with the same amount of protein, compared to the absorbance value in the presence or absence of DpdtbA, calculating the activity unit based on the formula provided by the company (Beyotime Biotechnology, China).

### 4.7. Western Blotting Analysis

Briefly, 1 × 10^7^ Kyse 450 (150 or 510) cells treated with or without the DpdtbA was scraped off in lysis buffer (50 mM Tris-HCl, pH 8.0, 150 mM NaCl, 1.0% NP-40, 10% glycerol and protease inhibitors), and the suspension was incubated on ice for 30 min. and then collected the clear supernatant by centrifugation at 14,000 ×g. A colorimetric Bio-Rad DC protein assay was employed to determine the protein concentration on a microplate reader MK3 at 570 nm. Proteins (20-30 *μ*g) were separated on a 13% sodium dodecyl sulfate-polyacrylamide gel at 200 V for 1 h. Then, the separated proteins were subsequently transferred onto a PVDF membrane at 60 V for 1 h. The membrane was washed three times with Tris-buffered saline (TBS) and was then blocked for 2 h in TBS containing 0.1% Tween-20 and 5% nonfat skimmed milk. The membrane was incubated at 4°C overnight with the primary monoantibody used at a dilution of 1 : 300 in TBS plus 0.1% Tween-20 (TBST). The membrane was washed several times with TBST and was subsequently incubated with HRP-conjugated secondary antibody (1 : 2,000 in TBST) for 2 h at room temperature. After another wash of the membrane with TBST, the protein bands were detected using a super sensitive ECL solution (Boster Biological Technology Co. Ltd.) and visualized on an SYNGENE G:BOX Chemi XX9 (SYNGENE, UK).

### 4.8. Statistical Analysis

Results are presented as the mean ± SEM. Comparisons between two groups were carried out using the two-tailed Student's *t*-test. Comparisons between multiple groups were performed by one way ANOVA with Dunnett's post hoc correction. A *p* value < 0.05 was considered statistically significant.

## Data Availability

The data used to support the findings of this study are included within the article.

## Supplementary Materials

Supplementary Materials Information on the effects of DpdtbA on cell growth, cell cycle, and ROS production. Other evidence suggested that the p53 downregulation induced by DpdtbA was not through ubiquitination but through stub1-mediated autophagy.
