# Supplementary material for: DpdtbA-Induced Growth Inhibition in Human Esophageal Cancer Cells Involved Inactivation of the p53/EGFR/AKT Pathway
Source: Oxid Med Cell Longev. 2019 Jul 1;2019:5414670. doi: 10.1155/2019/5414670 (PMC6636558; doi:10.1155/2019/5414670)
Supplement: Supplementary Materials — Information on the effects of DpdtbA on cell growth, cell cycle, and ROS production. Other evidence suggested that the p53 downregulation induced by DpdtbA was not through ubiquitination but through stub1-mediated autophagy. [file 5414670.f1.docx]

**Supplementary materials**

**DpdtbA inhibited colony formation in kyse150 cell line**

Similarly the effect of DpdtbA on colony formation in kyse150 cells was further investigated, clearly DpdtbA led to significant decrease in colony numbers (Figure S1A2), the quantitative analysis (Figure S1B) revealed that the inhibitory effect had statistics significance (p<0.05).

**
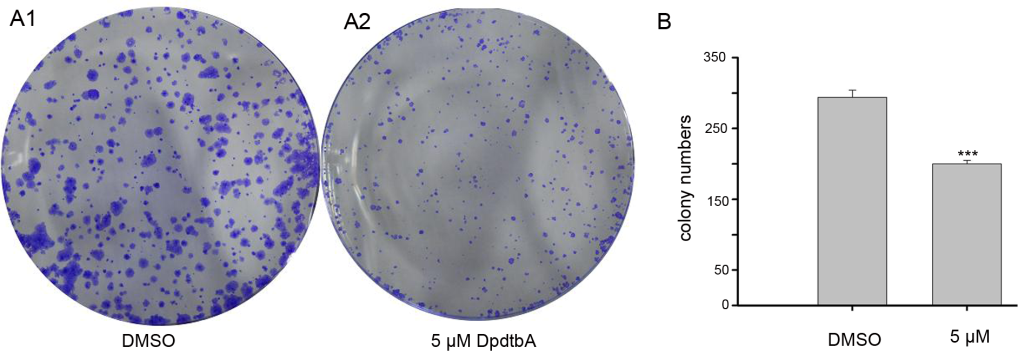
**

Figure S1: the effect of DpdtbA on colony formation in kyse 150 cells. (A): A1, DMSO; A2, 5 M DpdtbA. (B) Quantitative analysis of the effect of DpdtbA on colony formation (from two assays). ^***^ P<0.01.

**DpdtbA induced cell cycle arrest through CDK regulation**

Cyclins, CDK (cyclin dependent kinases), cyclin A and CDK2 are known to play an important role in the regulation of DNA synthesis during cell-cycle progression at S phase. The flow cytometry analysis revealed that DpdtbA led to S phase arrest in both investigated cell lines (Figure 1), the alteration of CDK2 level might contribute to the phase delay. To this end, the expression of CDK2 in different condition was determined. As shown in Figure S2, a down-regulated CDK2 was observed upon DpdtbA treatment, in accordance with that reported previously [see, reference 26 in main text].


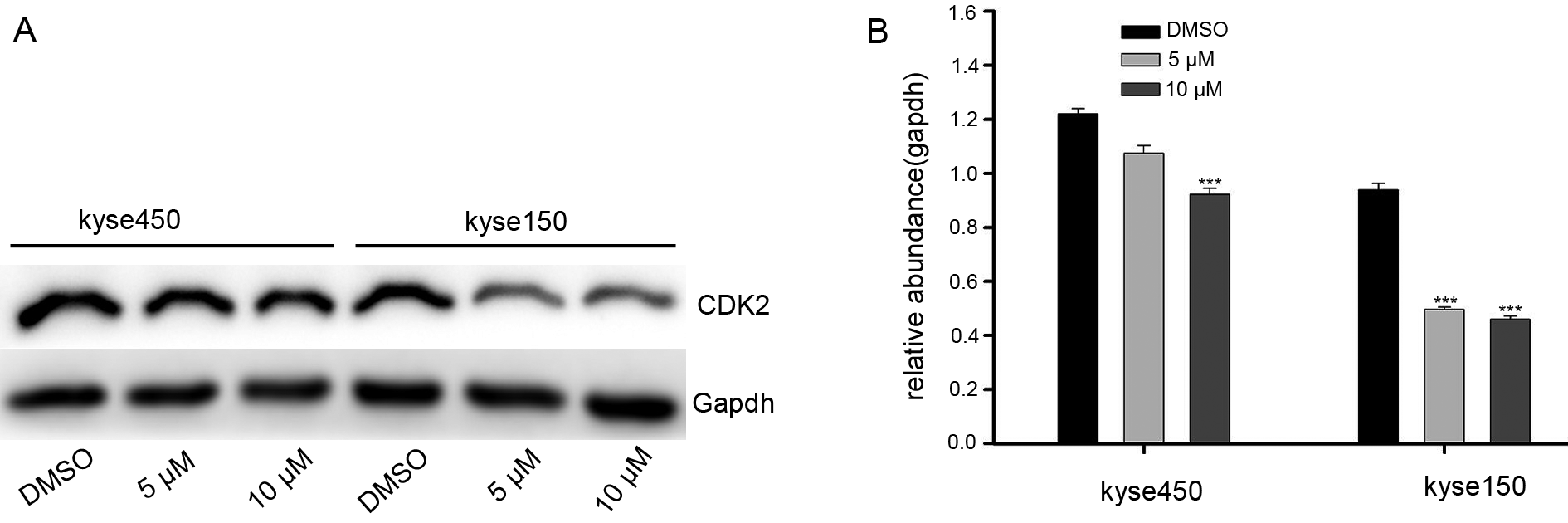


Figure S2: the effect of DpdtbA on CDK2 regulation. (A) Western blotting analysis of CDK2 in Kyse 450 and Kyse 150 cell; (B) quantification analysis of CDK2 (from twice measurements). The experimental condition was as indicated. ^***^P<0.01.

**DpdtbA induced DNA fragmentation.**

Kyse 450 (150) cells were first cultured in 6-well plate with cover glass overnight. Following DpdtbA treatment for 24 h, cells were first fixed with 4% paraformaldehyde in PBS for 20 min at 37°C, and then permeabilized with 0.2% triton-X-100 in PBS for 5 min. Following additional PBS washing, the cells were stained with DAPI (0.5 µg /ml), the fluorescent micrographs were captured using an inverted fluorescence microscope (Nikon, Tokyo, Japan). As showed in Figure S3, the DpdtbA induced nucleic DNA fragmentation in a concentration dependent manner, which led to increase of phosphorylated p53 (Figure 9).


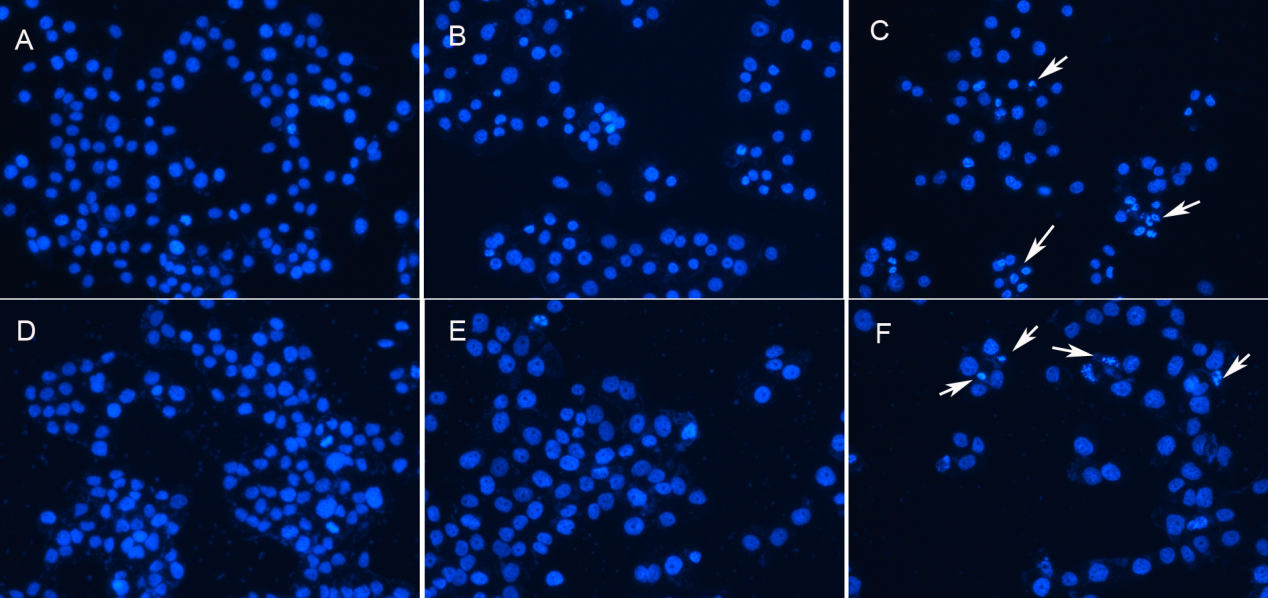


Figure S3: DpdtbA induced cellular DNA fragmentation in a concentration dependent manner. (A)-(C) Kyse 450: (A) DMSO; (B) 5 μM DpdtbA; (C) 10 μM DpdtbA. (D)-(F) Kyse 150: (A) DMSO; (B) 5 μM DpdtbA; (C) 10 μM DpdtbA. The arrow indicated the fragmentation of chromatins.

**DpdtbA induced apoptosis was detected by Acridine orange (AO)/Ethidium bromide (EtBr) stains**

To detect apoptosis an additional morphological assay was performed through acridine orange/ethidium bromide staining as reported previously [1]. Briefly, cells were grown on a coverslip and treated with DpdtbA for 24 hours, after which 10 μl AO/EtBr solution were added for 5 minutes. Then, the cells were washed twice with PBS and images were captured by a fluorescence microscope (Nikon, Tokyo, Japan). As shown in Figure S4, the cell populations with red fluorescence were increased with increase of DpdtbA in kyse 450 cells, indicating that more cells were in apoptosis (A-C). However this situation in kyse 150 cells was not obvious (D-F).


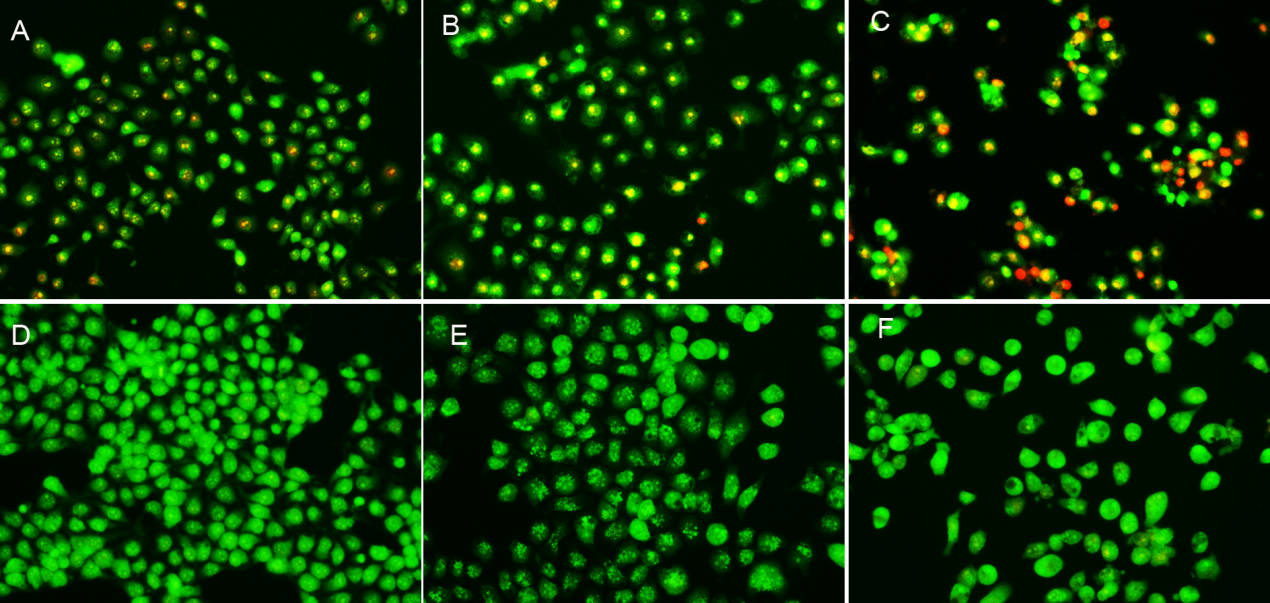


FIGURE S4: A DpdtbA induced apoptosis in ESC cells after 24 hours post-treatment as detected by EtBr/AO staining assay, A-C: kyse450 cell and D-F: kyse150 cell. (A, D) DMSO; (B, E) 5 µM DpdtbA; (C, F) 10 µM DpdtbA. Green, orange, and red fluorescence indicates live, apoptotic, and dead cells, respectively. Images were captured by fluorescence microscope (Nikon ECLIPSE TiE), object size: 20 × 10. AO, acridine orange; EtBr, ethidium bromide.

**DpdtbA induced ROS generation had less effect on mitochondrial membrane permeability**

As described above, Kyse 450 (150) cells were first cultured in 6-well plate, following DpdtbA treatment for 24 h, the cell culture was removed and washed with PBS. Next the cells were stained by rhodamine 123 for 30 min. at 37°C, following additional PBS washing, the fluorescent images were captured using an inverted fluorescence microscope (Nikon eclipse Ts2, Japan). Figure S5 showed that the accumulation of rhodamine 123, a mitochondrial dye, was not obviously increased, indicating the membrane of mitochondrial was in intact state, which was in accordance with results from Figure 5.


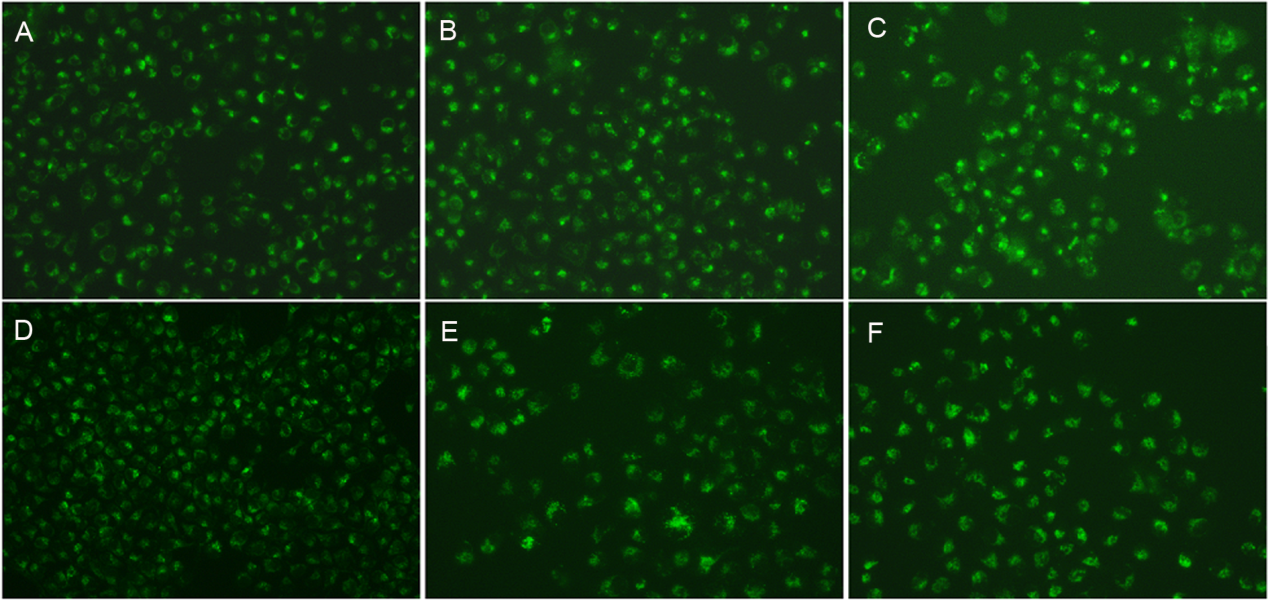


Figure S5: the effect of DpdtbA on mitochondrial membrane permeability. Rhodamine 123 was used for the assay. (A)-(C) Kyse 450: (A) DMSO; (B) 5 μM DpdtbA; (C) 10 μM DpdtbA. (D)-(F) Kyse 150: (A) DMSO; (B) 5 μM DpdtbA; (C) 10 μM DpdtbA.

**DpdtbA induced growth inhibition involved ROS production in kyse 150 cells**

Flow cytometry analysis revealed that the DpdtbA induced ROS production in Kyse150 cell was similar to that in kyse450 cell (Figure S6). The ROS production induced by DpdtbA was significantly increased compare to that in control (p<0.05).


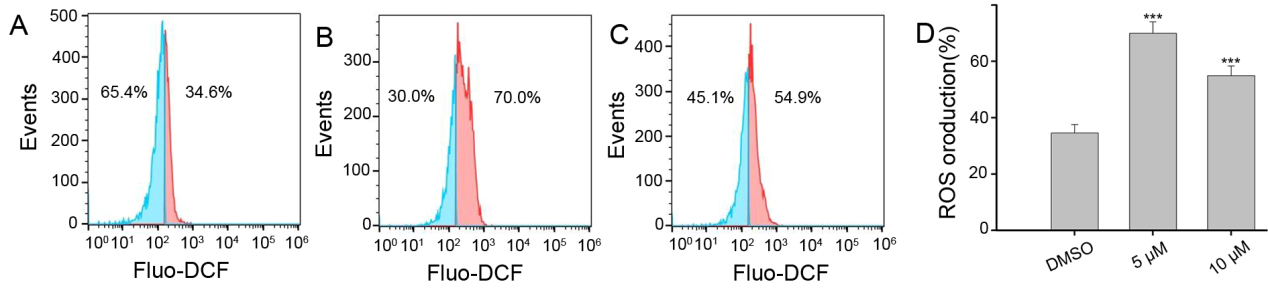


Figure S6: DpdtbA induced ROS generation in kyse 150 cells. (A) DMSO; (B) 5 µM DpdtbA; (C) 10 µM DpdtbA; (D) quantitative analysis of ROS production (from twice measurements). ^***^P<0.05.

**DpdtbA treatment led to upregulation of p53**

The Kyse 450 (Kyse 150) cells were treated with or without DpdtbA for 24 h in a six-well plate, following PBS washing, 1 ml of Trizol (Beyotime, China) was added to the plate to extract RNA as the company recommended. The reverse transcription was conducted as the company recommended. Briefly, 2 µg total RNA (A_260_/A_280_ ≥ 2.0) were added to a reaction buffer (total volume 20 µl), following addition of 1 µl random primer, finally 1 µl of MMLV reverse transcriptase (HiScript 1st Strand cDNA Synthesis Kit, Vazyme Biotech Co., Ltd, China) was introduced to initiate reaction of transcription (26 °C for 10 min, then 42 °C for 45 min; 75 °C for 10 min to kill the reverse transcriptase). The cDNA was stored at -80°C. 1 µl cDNA was used in all PCT reaction, primers for p53 were: forward 5’- GTCTACCTCCCGCCATAA -3’; reverse 5’- CATCTCCCAAACATCCCT -3’ and 18S as control, forward 5'-GCGGCGGAAAATA GCCTTTG-3’, and reverse 5'-GATCACACGTTCCACCT CATC-3'. PCR condition: 35 cycles of amplification at 95°C for 30 sec, 45°C for 30 sec, and 72°C for 1 min. RT-PCR products were examined by agarose gel electrophoresis. After ethidium bromide staining, bands were visible only at the expected molecular weights for the p53 mRNA and internal control products on a Tocan 360 scanner (Shanghai Tiancheng Technology Co., Ltd. China).


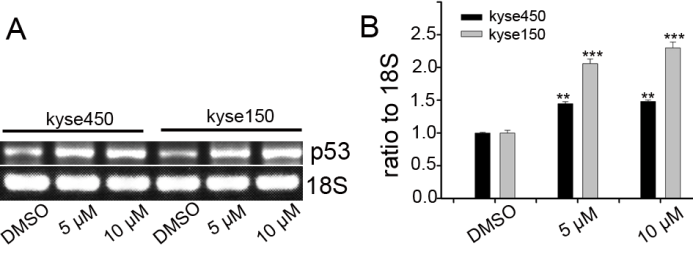


Figure S7：The effect of DpdtbA on TP53 gene expression in transcription. (A) RT-PCR products after electrophoresis. (B) Quantitative analysis of p53 before and after DpdtbA treatment (from trice measurements). ^**^P<0.05, ^***^P<0.01.

**MDM2 knockdown via small-interfering RNA (Si-RNA) did not increase p53 expression**

The small-interfering RNA (Si-RNA) to knockdown MDM2 was conducted to further confirm that the p53 degradation was not through ubiquitination. Briefly, after removing culture and washing with PBS, Kyse 450 cells (1×10^6^) were transfected with 100 pmol of siRNA using Lipofectamine™ Stem Transfection Reagent (Invitrogen, USA) for 12 h as the manufacturer’s recommended protocol. Next the DpdtbA was added to the cells with complete medium for 24 h incubation at 37℃ in a humidified atmosphere of 5% CO_2_. The Western blotting analysis is shown in Figure S8, clearly down-regulation of MDM2 did not attenuate depletion of p53, indicating that p53 degradation was not through ubiquitination.


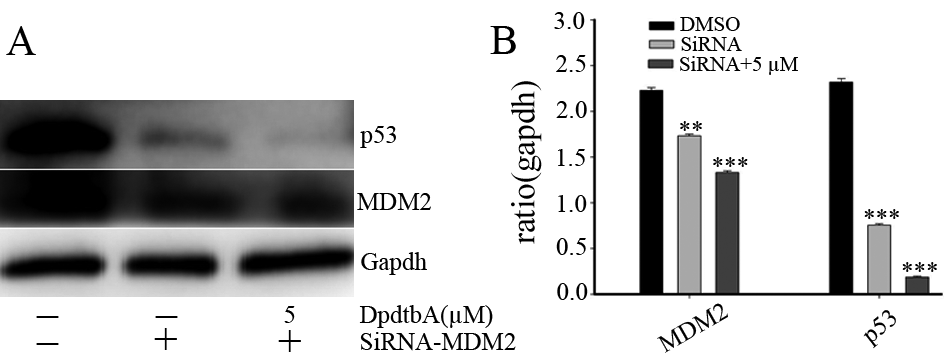


Figure S8：MDM2 knockdown by siRNA did not increase p53 expression. (A) western blotting analysis of p53 and MDM2 in Kyse 450 cell; (B) quantification analyses of MDM2 and p53 (from twice measurements). ^**^P<0.05, ^***^P<0.01.

**Ubiquitination was not responsible for DpdtbA induced p53 depletion**

As shown in Figure S9, the PFT-α did not obviously decrease the p53 expression, which was consistent with that reported previously due to p53 mutation the Kyse 450 cell. Interestingly the proteasome inhibitor, MG132 did not restore the p53 level to that in control, indicating that ubiquitination was not responsible for DpdtbA induced p53 depletion. Meanwhile the iron addition abolished the action of DpdtbA, indicating that the chelating behavior of DpdtbA had role in induced growth inhibition.


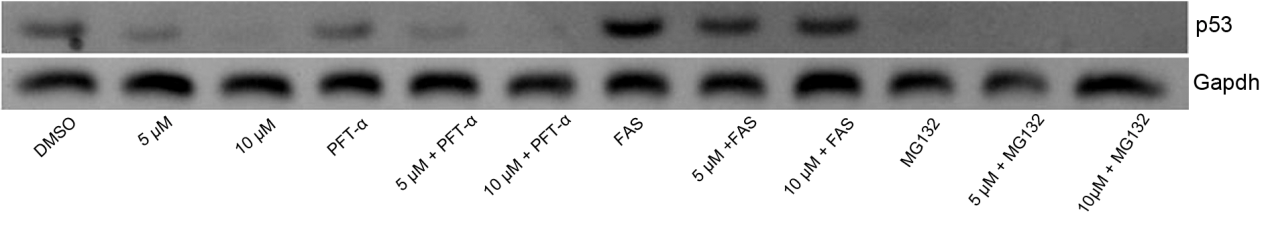


Figure S9: proteasome degradation was not involved in DpdtbA induced p53 depletion. The condition was as indicated in the figure. FAS = (NH_4_)_2_Fe(SO_4_)_2_

**Stub1 was responsible for autophagic degradation of p53**

Since MDM2 did not involve p53 degradation, the autophagy may be responsible for p53 downregulation, the Western blotting analysis clearly showed that DpdtbA induced a downregulation of Stub1, suggesting a autophagic degradation of p53 occurred. To support the conclusion, Stub1 knockdown by siRNA was further conducted. As shown in Figure S10, siRNA-stub1 decreased both stub1 and p53 expression, supporting that p53 degradation was associated with stub1, in accordance with result from literature [2].


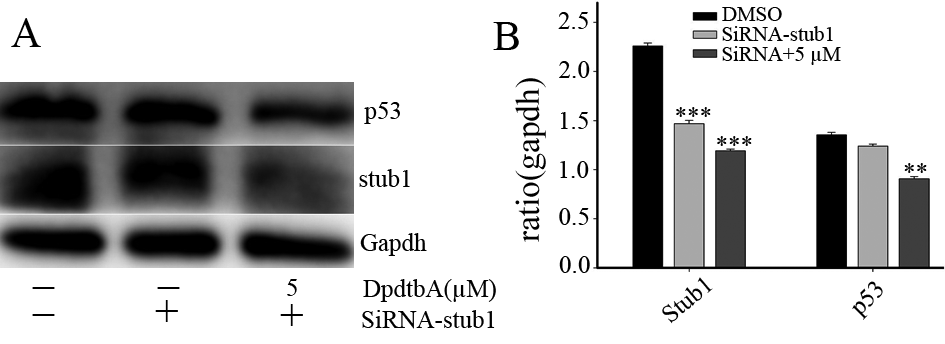


Figure S10: Stub1 knockdown by siRNA led to p53 depletion as like DpdtbA. (A) Western blotting analysis of p53 and stub1in Kyse 450 cell; (B) quantification analyses of stub1 and p53 (from twice measurements). ^**^P<0.05, ^***^P<0.01.

**Reference**

1. K. Liu, P.C. Liu, R. Liu and X. Wu, "Dual AO/EB staining to detect apoptosis in osteosarcoma cells compared with flow cytometry," Medical Science Monitor Basic Research, vol. 21, no. 1, pp. 15-20, 2015.
2. H. Vakifahmetoglu-Norberg, M. Kim, H. G. Xia, et al., “Chaperone-mediated autophagy degrades mutant p53,” Genes & Development, vol. 27, no. 15, pp. 1718-1730, 2013.
